# Expression of Concern: LncRNA DANCR promotes migration and invasion through suppression of LncRNA-LET in gastric cancer cells

**DOI:** 10.1042/BSR-20171070_EOC

**Published:** 2020-07-29

**Keywords:** DANCR, EZH2, HDAC3, lncRNA-LET

The Editorial Office has been made aware of potential issues surrounding the scientific validity of this paper, hence has issued an expression of concern to notify readers whilst the Editorial Office investigates.

